# Functional roles of fibroblast growth factor receptors (FGFRs) signaling in human cancers

**DOI:** 10.1007/s10495-013-0886-7

**Published:** 2013-07-31

**Authors:** Kai Hung Tiong, Li Yen Mah, Chee-Onn Leong

**Affiliations:** 1 School of Postgraduate Studies and Research, International Medical University, Bukit Jalil, 57000 Kuala Lumpur, Malaysia; 2 School of Pharmacy, International Medical University, Bukit Jalil, 57000 Kuala Lumpur, Malaysia; 3 Center for Cancer and Stem Cell Research, International Medical University, 126 Jalan 19/155B, Bukit Jalil, 57000 Kuala Lumpur, Malaysia

**Keywords:** Fibroblast growth factor receptors, Cancer, Signal transduction, Targeted therapy

## Abstract

The fibroblast growth factor receptors (FGFRs) regulate important biological processes including cell proliferation and differentiation during development and tissue repair. Over the past decades, numerous pathological conditions and developmental syndromes have emerged as a consequence of deregulation in the FGFRs signaling network. This review aims to provide an overview of FGFR family, their complex signaling pathways in tumorigenesis, and the current development and application of therapeutics targeting the FGFRs signaling for treatment of refractory human cancers.

## Introduction

The human fibroblast growth factor receptor (FGFR) family, a subfamily of receptor tyrosine kinases (RTKs), comprises of four family members—FGFR1, FGFR2, FGFR3 and FGFR4. A closely-related receptor which lacks the FGF signaling tyrosine kinase domain, FGFR5, (also known as FGFRL1) was recently discovered on the basis of interaction with FGFR-binding ligands, known as fibroblast growth factors (FGFs) [1]. Collectively, FGFR signaling is associated with the activation of multiple cellular cascades and responses such as cell growth, proliferation, differentiation, and survival [2–4].

## FGFR protein structures

**Fig. 1:**
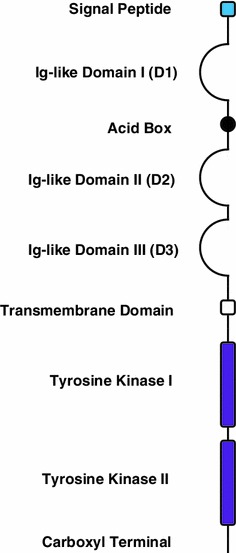
The basic structure of a FGFR. The FGFRs are phylogenetically closely related to the VEGFRs and PDGFRs, consist of three extracellular immunoglobulin (Ig) domains (D1-D3), a single transmembrane helix, an intracellular split tyrosine kinase domain (TK1 and TK2) and an *acidic box*. D2 and D3 form the ligand-binding pocket and have distinct domains that bind both FGFs and heparan sulfate proteoglycans (HSPGs). *Acidic box* is required for binding of bivalent cations for optimal interaction between FGFRs and HSPGs

The FGFR family members share a high percentage of sequence homology (~55–72 %) [5], and consist of three important domains—extracellular ligand-binding domain, single transmembrane domain, and intracellular tyrosine kinase domain (Fig. 1). The extracellular ligand binding-domain comprises of a hydrophobic signal peptide region and two or three immunoglobulin (Ig)-like domains, designated D1–D3. The acid box is made up of ~30 serine residues and connects D1 and D2. The transmembrane domain facilitates signal transduction from the extracellular region into the cytoplasmic domain. Emerging from the cytoplasmic membrane is a juxtamembrane region, followed by a split tyrosine kinase domain, and finally a COOH tail [2, 5, 6].

Although FGFR5 is structurally similar to other FGFRs, it lacks the intracellular protein tyrosine kinase domain, which is replaced by a short intracellular tail with a histidine-rich motif [1]. Due to the absence of the tyrosine kinase domain, FGFR5 cannot signal by transautophosphorylation (as other FGFRs), and hence does not function like other FGFRs. Instead, FGFR5 is proposed to act as decoy receptor that binds FGF ligands and sequesters them away from the conventional FGFRs [1]. Additionally, the signal peptide found within the extracellular ligand binding-domain is cleaved off upon insertion into the endoplasmic reticulum, and FGFR5 is occasionally shed from the plasma membrane and is found in a secreted, soluble form [1]. FGFR5 binds to certain FGFs, as do the conventional FGFRs, but it does not bind to FGF1, which is also recognized by all of the other FGFRs [1].

## Alternative splicing of FGFRs

**Fig. 2:**
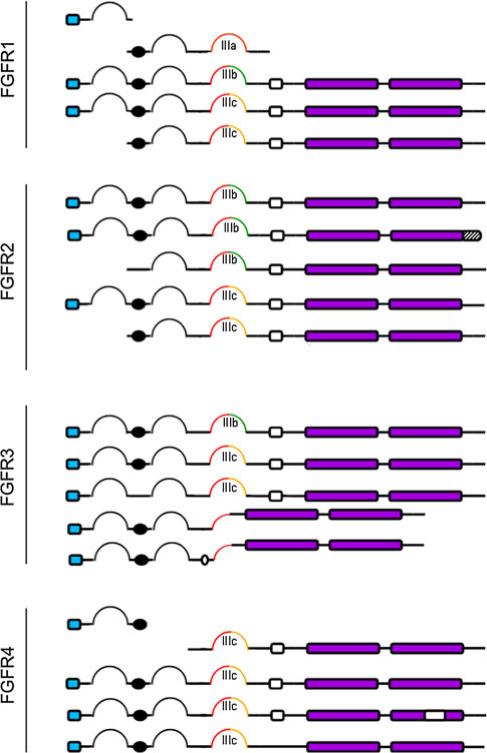
FGFR splice variants. The FGFRs isoforms are generated mainly by alternative splicing of the Ig III domain (D3). The D3 could be encoded by an invariant exon 7 (*red*) to produce FGFR-IIIa isofom or spliced to either exon 8 (*green*) or 9 (*yellow*) to generate the FGFR-IIIb or FGFR-IIIc isoforms, respectively. Epithelial tissues predominantly express the IIIb isoform and mesenchymal tissues express IIIc. FGFR4 is expressed as a single isoform that is paralogous to FGFR-IIIc. *Hatched box* represents a truncated carboxyl terminal. *Clear box* indicates a deletion of an exon

Despite the general characteristics shared among the family members of FGFRs, an array of isoforms exist within each family member (Fig. 2). Structural diversity observed across the isoforms of FGFRs, is largely attributed to the alternative splicing of endogenous mRNA sequence. These isoforms include the secreted form of FGFRs that lack the hydrophobic membrane-spanning region and the entire cytoplasmic catalytic domain [7, 8], FGFRs with an extracellular domain composed of either two or three Ig-like domains [7, 9–11], and FGFRs devoid of the acid box [12].

One of the most important mechanisms that determine the ligand-binding specificity of FGFRs is by alternate exon usage of the IgIII (D3) domain to produce three possible IgIII domains isoforms, designated IgIIIa, IgIIIb and IgIIIc. IgIIIa is encoded entirely by exon 7 alone while IgIIIb and IgIIIc are derived from alternative splicing of exon 7/8 and exon 7/9, respectively (Fig. 2) [13–16]. The IgIIIb and IgIIIc splice variants are commonly observed in *FGFR1*, *FGFR2* and *FGFR3* gene [13, 17]. The *FGFR4* gene is unique as only IgIIIc variants are present [18]. FGFR5 isoforms lacking the sequences that encode for either the first Ig domain or the first Ig domain plus the acidic box were also identified, but the properties of these isoforms are not known and no tissue-specific expression has yet been reported [1].

## Activation and signaling of FGFR

**Fig. 3:**
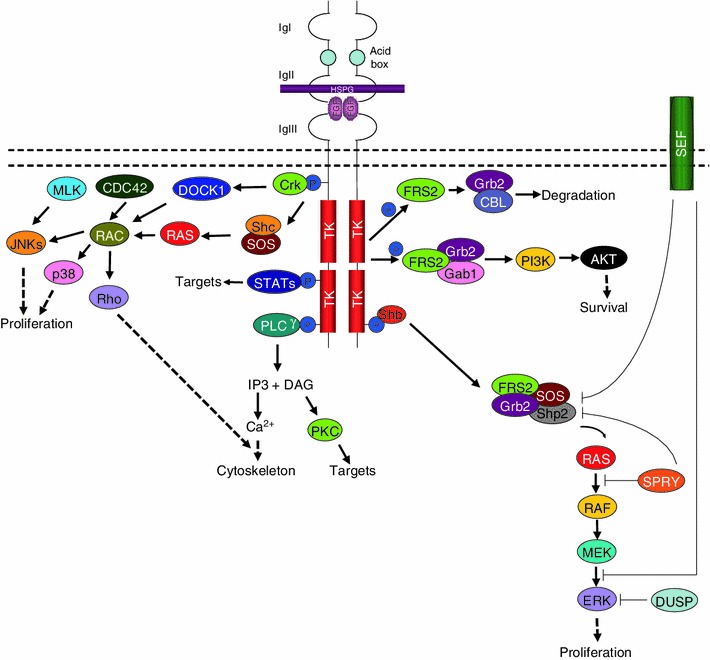
FGFR signaling pathway. FGFs induce FGFR-mediated signaling pathway by interacting with specific FGFRs and HSPGs. The macromolecular interactions mediate FGFRs dimerization or oligomerization and activate multiple signal transduction pathways, including those involving FRS2, RAS, p38 MAPKs, ERKs, JNKs, Src, PLCγ, Crk, PKC and PI3K. These pathways are negatively regulated in part by the activities of DUSPs, SPRY, SEF and CBL

FGFR signaling is primarily triggered by the binding of the receptors to FGF ligands (Table 1), and the subsequent formation of various complexes to initiate downstream signal transduction including activation of PLCγ, MAPK, AKT, and STAT cascade (Fig. 3) [19].

**Table 1:** Ligand specificity of the fibroblast growth factor receptor family [188–201]

FGFR isoform	Ligand specificity
FGFR1, IIIb	FGF-1, 2, 3, 10, and 22
FGFR1, IIIc	FGF-1, 2, 4, 5, 6, 19, 20, and 21
FGFR2, IIIb	FGF-1, 3, 4, 6, 7, 10, and 22
FGFR2, IIIc	FGF-1, 2, 4, 5, 6, 8, 9, 17, 18, 19, 21, and 23
FGFR3, IIIb	FGF-1 and 9
FGFR3, IIIc	FGF-1, 2, 4, 8, 9, 17, 18 19, 21, and 23
FGFR4	FGF-1, 2, 4, 6, 8, 9, 16, 17, 18, and 19

The phosphotyrosine residues in the carboxy-terminal regions of FGFR confer selective and strong binding to Src homolog 2 (SH2) domain-containing molecules, such as phospholipase Cγ (PLCγ) [20, 21]. This interaction results in the hydrolysis of phosphatidylinositol 4,5-biphosphate (PIP_2_) to generate two effectors, inositol 1,4,5-triphosphate (IP_3_) and diacylglycerol (DAG) [22]. Accumulation of IP_3_ further stimulates calcium release from internal stores, whereas DAG mediates the activation of protein kinase C (PKC) and other downstream targets such as the Ras/MEK/MAPK pathway (Fig. 3) [22].

One of the many adaptor proteins which facilitates signal transduction from FGFRs is the v-crk sarcoma virus CT10 oncogene homolog (avian) (Crk). Upon exposure to growth factors, the juxtamembrane tyrosine residue 463 of FGFR1 is phosphorylated, followed by the transient phosphorylation of Crk to mediate the co-complexing of FGFR1 and Crk [23]. Crk has been reported to associate with various signaling molecules such as the guanine nucleotide exchange factor, SOS [24] and C3G [25], as well as the dedicator of cytokinesis 1 (DOCK1) [26]. SOS then activates JNK via Ras [27] and Rac [28, 29]. In addition to Rac, cdc42 has also been proposed as an intermediate to the JNK and p38 activation cascades [27]. Direct interaction of DOCK180 to Rac1 have been reported, and DOCK180 activates JNK in a manner dependent on Cdc42Hs, and SEK [30]. On the other hand, C3G activates JNK1 through a pathway involving the MLK family of protein [31].

Activation of FGFRs also lead to phosphorylation of the docking protein FGFR substrate 2 (FRS2) followed by the recruitment of Shp2 tyrosine phosphatase, whereby the subsequent phosphorylation of Shp2 facilitates its association with growth factor receptor-bound 2 (Grb2) and SOS [32–35]. This complex triggers the induction of the Ras/MEK/MAPK signaling pathway [32]. In addition, the tyrosine phosphorylation of FRS2α also mediates the recruitment of Grb2 and Gab1, resulting in the recruitment and activation of PI3-kinase [36]. Alternatively, the Grb2/FRS2α complex interacts with Cbl via the SH3 domains resulting in the ubiquitination of FGFRs and FRS2α for the attenuation of growth factor signaling [37].

Accessory proteins such as SH2 domain-containing adaptor protein B (Shb) and SH2 domain-containing collagen (Shc) also interact with FGFRs to facilitate signal transduction [22, 38–43]. Shb2 binds to tyrosine 766 in FGFR1 to facilitate the phosphorylation of FRS2 for the subsequent activation of the Ras/MEK/MAPK pathway [22]. Similar to Shp2, Shc is an intermediate molecule which mediates the assembly of FGFR and Grb2-SOS complexes, also to activate the Ras/Raf/MEK/MAPK pathway [3, 41, 44]. Shc co-localizes exclusively with FGFR2 at the plasma membrane and intracellular membranous compartment [45]. The localization of Shc and the stabilization of the binding of its SH2 domain to FGFR2 is attributed to the presence of the phosphotyrosine-binding (PTB) domain [45, 46].

FGFR also binds to signal transducers and activators of transcription (STAT) and ribosomal protein S6 kinase 2 (RSK2). The interaction between FGFR and STAT was first observed in polymorphism studies, in which the point mutation—K650E of FGFR3, and other FGFR isoforms, led to the constitutive activation of both FGFR and STAT [47–49]. STAT3 was further documented to bind to phosphorylated Tyr^677^ of FGFR1 [50]. In addition, tyrosine activation of STAT3 requires the overexpression of FGFR1 or FGFR2 (Fig. 3) [50].

## Negative regulation of FGFR signaling

Given the fact that FGFRs activate an array of signaling pathways, it is crucial to have a regulatory system in place to circumvent unnecessary signal transmission. FGF signaling is negatively modulated by regulators such as Cbl proto-oncogene E3 ubiquitin protein ligase (CBL), sprouty homolog (SPRY), similar expression to fgf genes (SEF) and MAPK phosphatases (MKP) (Fig. 3).

Following activation, FGFRs are often ubiquitinated by CBL to facilitate clathrin-mediated endocytosis and receptor degradation [37, 51]. Intriguingly, different FGFR isoforms have discrete propensities for ubiquitination. FGFR1 is more susceptible to ubiquitination whereas FGFR4 has only a few lysine ubiquitination sites [52, 53]. The difference in ubiquitination is thought to account for the different sorting of FGFRs to the lysosome [52].

A second pathway that negatively regulates FGFR signaling involves the inhibition of RAS/RAF/ERK downstream pathway through SPRY [54–59]. Upon FGF ligand stimulation, SPRY isoforms form homo- and hetero-oligomers via their C-terminal domains and attenuate ERK activation through interaction with FRS2–Grb2-SOS complex or direct inhibition of RAS/RAF signaling pathway (Fig. 3) [56, 60–64]. Among all the SPRY isoforms, SPRY2 is considered more inhibitory than SPRY1 and SPRY 4 [61], but the SPRY1/SPRY4 hetero-oligomer exhibits the most potent inhibitory effect on ERK [60]. However, the binding of SPRYs to Grb2 does not always result in ERK inhibition [65, 66]. This implies that SPRY can inhibit signal transduction in a Grb2-independent pathway, and the molecular machinery that is involved in this context is worth investigating. In this context, it is likely that the functional role of SPRY in FGFR signaling is highly dependent on the cell type and stimuli, and might play a different role in different cell types.

In addition to CBL and SPRY, SEF has also been shown to negatively modulates FGF-mediated ERK and AKT activation [67–70]. In addition to receptor inhibition, SEF could also hinder the nuclear translocation of ERK in certain cell lines (Fig. 3) [71, 72].

The role of dual phosphatases in the negative feedback of growth factor signaling has also been reported by many researches. For instance, the MAP kinase phosphatase -1 (MKP-1) has been discovered on the basis of its ability to dephosphorylate MAP kinase in vivo [73]. Similarly, MKP-3 blocks both the phosphorylation and enzymatic activation of ERK2 by mitogens [74]. Consistent with this, the targeted inactivation of *Dusp6*, which encodes MKP-3, led to the increase of phosphorylated ERK and pERK targets [75]. However, not every member of this class of dual phosphatase inhibits ERK. Although MKP-3 blocks ERK1 activation by oncogenic p21(ras), but ERK1 activated by p21(ras) (G12V) is insensitive to the M3/6 dual phosphatase [76].

## FGFRs and human cancer

Numerous human pathological conditions are tightly associated with the deregulation of FGFR signaling. Aberrant FGFR signaling is largely attributed to several underlying mechanisms involving gene amplification, single nucleotide polymorphism (SNP), chromosomal translocation, ligand availability and impaired termination program in FGF-mediated signaling, which is reviewed in the following section. In addition, a further layer of complexity is added by the fact that FGFRs are subjected to alternative splicing, giving rise to multiple isoforms which may promote or repress tumorigenesis, under different circumstances.

### Gene amplification

Enhanced FGFR expression is commonly observed in various types of human malignancies. Such elevations in FGFRs expression could be due to gene amplification or deregulation at the transcriptional level (Table 2) [77–80]. In line with this, the chromosomal regions 8p11-12 and 10q26 are consistently amplified in human breast cancers, and some of the genes within this region that are amplified are FGFR1 and FGFR2 [77–83]. Amplifications of 8p11 and 8p12 are associated with early relapse, poor prognosis and survival, especially in ER-positive breast cancer patients where FGFR1 amplification drives resistance to hormonal therapy [84–86]. Although high copy numbers and expression levels of FGFR1 has been shown to promote tumorigenesis [79, 87–90], several studies also show that amplification of the focal region 8p11-12 does not always result in the overexpression of FGFR1 [91–93]. In this case, other genes located at the same locus may also be amplified and contribute to the oncogenesis of human mammary carcinomas [92], independent of FGFR1. Indeed, a few candidate genes identified from the recurrent amplicon 8p11-12 were overexpressed and promotes the survival of the breast tumors [93].

**Table 2:** Gene amplifications of FGFRs in human cancers

FGFRs	Cancer types	References
FGFR1	Breast, ovarian, bladder, lung and rhabdomyosarcoma	[77–83, 86, 201–206]
FGFR2	Gastric, breast, lung, endometrial and esophageal cancer	[78, 80, 82, 95–102, 207–211]
FGFR3	Bladder and salivary adenoid cystic cancer	[212, 213]
FGFR4	Gastric, breast and ovarian cancer	[80, 83, 94, 214]

Amplification of the FGFR2 gene is identified in gastric, lung, and endometrial primary tumors and biologically-transformed cell lines [19, 78, 80, 82, 83, 94–102]. Furthermore, this event is occasionally accompanied by other forms of genetic alteration such as base deletion. For example, scirrhous-type gastric cancer cells which harbor amplification in the region 10q26, were also found to contain deletions in the C terminal of the FGFR2 exons [103]. It is postulated that the presence of highly amplified copies of the truncated receptor could promote tumor proliferation and oncogenesis [104–106].

It is also common to observe secondary chromosomal locus amplification in parallel to elevated FGFR expression. For instance, 30–40 % of breast tumors with amplification of the FGFR1-containing chromosomal region 8p12, were concurrently presented with CCND1 (Cyclin D1) gene amplification at the 11q13 locus [77]. Since the number of genetic alterations observed in tumor is associated with poor prognosis [107], there could be a synergistic effect from the concomitant amplification of both oncogenes to drive tumorigenesis.

### Point mutations

Various human diseases are driven, in part, by point mutations. The sequencing of cancer genomes has uncovered over a thousand somatic mutations in the coding exons of 518 human kinase genes [108]. Most of the non-synonymous mutations involve FGF signaling [108]. Oncogenic point mutations of FGFRs could essentially include any parts of the receptor, as summarized in Table 3. Some of these mutations have been shown to exert a gain-of-function effect, contributing to the developmental abnormalities, uncontrolled growth, and metastasis in a various cancerous cells, the role of other mutations in tumorigenesis remained unknown [108].

**Table 3:** Point mutations of FGFRs in human cancers

FGFR	Cancers	Mutations	References
FGFR1^a^	Lung	G70R^e^, T141R^e^, P252T/S, P576H, V664L	[215, 216]
	Prostate	R78H	[217]
	Breast	S125L	[108, 218, 219]
	Skin	P252T/S	[108, 217, 220]
	Stomach	A268S	[217]
	Colon	A268S, A429S	[217, 221]
	Brain	N546K^f^, R576W, K656E^f^	[222]
FGFR2^b^	Skin	S24F, V77M, E160A, H213Y, E219K, G227E, V248D, R251Q, G271E, G305R, T370R, W474X, E475K, D530N, E574K, E636K, M640I, I642V, A648T, S688F, G701S, P708S, R759X/Q, L770V	[223]
	Bladder	M71T	[217]
	Lymphoma	M71T	[217]
	Cervix	A97T	[115]
	Endometrial	D101Y, S252W^f^, P253R^f^, K310R, A314D, A315T, S372C, Y375C, C382R, A389T, M391R, I547, N549, K659/M/E	[115–117, 201, 224]
	Breast	R203C	[108, 218, 225]
	Lung	N211I, D283, W290C, I380, H544Q^e^, R612T^g^	[108, 115, 201, 216, 220, 226]
	Brain	Q212, G462E	[227, 228]
	Ovary	S252W^f^, G272V, Y375C	[229]
	Stomach	S267P^f^	[230]
	Colon	Q361R^h^, P582L	[228]
FGFR3^c^	Lung	T79S	[108]
	Multiple myeloma	G197S^e^, Y241C, R248C^f^, P250R^e^, Y373C^f^, G380R^f^, G382D^f^, F384L^e^, S433C, A441T^e^, A452S^e^, K650E/Q/M/N/T^f^, A717T^e^, I726F^e^	[137, 201, 231–239]
	Colon	C228R, E322	[108, 230]
	Bladder	R248C, S249C, G370C, S371C, Y373C, I376C, G380R, G382D, F384L^e^, A391E, D646Y, K650E/Q/M/N/T	[120, 201, 240–250]
	Head and neck	R248C^f^, S249C^f^, D617G, V630, E686, G697C^f^	[251–253]
	Cervix	S249C^f^	[119, 254, 255]
	Prostate	S249C^f^, F384L^e^, A391E^f^	[256]
	Brain	E466	[257]
	Testis	K650E/Q/M/N/T^f^	[258]
FGFR4^d^	Rhabdomyosarcoma	C56S, R72L, T122A, A175T, R234H, G388R^e^, N535/D, V550E^f^/L/M^i^, A554, G576D	[259]
	Lung	R183S^e^, S232I^e^, G388R^e^, R616G^e^, E681, P712T^g^, A729G^e^, S772	[108, 127, 201, 216, 217, 220, 260–262]
	Breast	Y367C^f^, G388R^e^, V550E^f^/L/M^a^	[108, 124, 217, 218, 263]
	Stomach	G388R^e^	[264]
	Skin	G388R^e^, P716R	[217, 223, 265]
	Brain	G388R^e^	[217]
	Colon	G388R^e^	[124]
	Liver	G388R^e^	[266]
	Soft tissue sarcoma	G388R^e^	[267]
	Prostate	G388R^e^	[217, 263, 268–271]
	Head and neck	G388R^e^	[217, 272–274]

^a^Mutations relative to the FGFR1 IIIc (GenBank accession number NM_023110)

^b^Mutations relative to the FGFR2 IIIc (GenBank accession number NP_000132)

^c^Mutations relative to the FGFR3 IIIc (GenBank accession number NP_000133)

^d^Mutations relative to the FGFR4 (GenBank accession number X57205)

^e^Single nucleotide polymorphism (SNP)

^f^Gain-of-function mutations have been demonstrated experimentally

^g^R612T is referred to as R496T in the literature due to a numbering relative to FGFR2 isoform 7 precursor which lacks two exons compared with transcript variant 1

^h^Glu361 is only present in FGFR2 IIIb

^i^FGFR4 V550M, P712T and S772N are referred to as V510M, P672T and S732N (respectively) in the literature and in COSMIC due to a numbering relative to FGFR4 transcript variant 2, which lacks 40 amino acids (including the transmembrane domain) compared with X57205

Various hotspots for point mutations have been characterized in FGFR2. Substitution of S252W and P253R alter the ligand binding specificities of FGFR2b and FGFR2c, but retain the ligand-dependent activation properties [109], whereas K659N causes receptor hyperactivation [110]. In terms of disease pathogenesis, point mutations of S252W and P253R in FGFR2 are associated with Apert syndrome [111], and some of these led to a crouzonoid phenotype [111–114]. Similar point mutations were also observed in various cancer types, such as uterine carcinoma and endometrial carcinoma [115–117], suggesting that point mutations which causes skeletal disorders are also causally linked to tumorigenesis.

In addition, activating mutant forms of FGFR3 attributed to point mutations are frequently detected in bladder cancer [118–120]. The substitution of cysteine residues at the extracellular domain and juxtamembrane region enhances intermolecular disulfide bonding and ligand-independent receptor dimerization [121–123]. These events eventually led to the continuous activation of FGFR3 and its downstream signaling pathways.

FGFR4 G388R is one of the most common nucleotide polymorphisms (SNPs), with at least one copy identified in nearly 50 % of the population [124]. G388R sustains the activation of FGFR4 [125], and human cancers characterized by this SNP were reported to be highly aggressive and metastatic in nature [124, 126–129]. Additionally, the FGFR4 R388 allele is also associated with invasion and metastasis by stabilizing the endosomal MMP14, thus promoting collagen degradation [126, 130]. The elevated expression of MMP1 further stimulates the autophosphorylation of FGFR4 R388, and collectively these regulators act synergistically to promote tumor invasion and metastasis [126, 130].

### Chromosomal translocation

Fusion proteins arise from intragenic chromosomal rearrangements that commonly involve reciprocal translocations. As a result of chromosomal fusions, the mutated proteins gain new functions which dominate the functions of wild-type proteins. Human FGFR fusion proteins generally consist of two main segments—the anterior being a dimerized domain from a partnering gene and tyrosine kinase domain at the posterior [131]. Unlike wild type receptors, mutant FGFRs are expressed intracellularly and retained in the cytosol, thus they escape the typical receptor degradation processes, further prolonging the activation signal [3].

FGFR-related fusion proteins which are caused by intragenic chromosomal translocation have been detected in hematological cancers (Table 4). These fusion proteins are mainly found in patients diagnosed with stem cell leukemia lymphoma (SCLL) (also known as 8p11 myeloproliferative) syndrome. FGFR fusion proteins are able to transform normal cell lines into SCLL or chronic myelogenous leukemia-like disease (CMLL) [132–136]. Interestingly, newly diagnosed multiple myeloma (MM) patients that harbor the t(4;14) translocation often exhibit overexpression of FGFR3 in the absence of activating mutations, while late stage MM patients carrying the same translocation were observed to have activated FGFR3 and multiple myeloma SET domain (MMSET) mutations [137]. It is believed that MMSET might contribute to cellular adhesion, clonogenic growth, and tumorigenicity [138]. The mechanism which drives the change in FGFR3 activation status during the early and late onset of MM has yet to be elucidated. One plausible explanation is that other forms of mutations that accompany FGFR3 translocation may trigger and maintain the abnormal signaling of FGFR3 [137].

**Table 4:** Fusion proteins of FGFRs found in human cancers

FGFRs	Fusion partners	Cancers
FGFR1	ZNF198/RAMP/FIM/ZMYM2^a^	SCLL [275–281]
	FOP/FGFR1OP1^a^	SCLL [282–284], lung [285]
	CEP110/CEP1^a^	SCLL [284, 286–289]
	BCR	SCLL [136, 290–293]
	LRRFIP1	SCLL [294]
	FGFR1OP2^a^	SCLL [295], AML [296]
	TRIM24/TIF1	SCLL [297]
	MYO18A	SCLL [298]
	CPSF6	SCLL [299]
	HERV-K	SCLL [300]
	PLAG1	H&N [301]
	CUX1	L/EMS/L [302]
	TACC1	Glioblastoma [303]
	FOXO1	Rhabdomyosarcoma [304]
FGFR3	TEL/ETV6^a^	T-cell Lymphoma [305]
	TACC3^a^	Glioblastoma [303], bladder [306]

^a^Fusion proteins which have been demonstrated to be functionally oncogenic in cell lines and mouse models

### Addiction to FGF ligand via autocrine and paracrine signalling

Soluble mitogenic growth factors can be synthesized by one cell type and signals the proliferation of another cell type, and the intersignaling between two different cells are termed paracrine signaling [139]. Alternatively, cancer cells may also produce growth factors to which they are responsive, creating a positive feedback signaling loop often termed autocrine stimulation [139].

One example of autocrine FGF-signaling is the elevated expression of FGFR1 and its high-affinity ligand, FGF2 in human melanoma xenografts. The silencing of either FGFR1 or FGF2 arrests tumor growth, suggesting the presence of the FGFR1–FGF2 autocrine loop in the subset of human subcutaneous carcinomas [140]. Similarly, in multiple basal-like breast cancer cells, tumor growth was suppressed following RNAi-mediated silencing of endogenous FGF2 [141]. In human non-small-cell lung carcinomas (NSCLC), FGF2, FGF9, and their respective receptors, were also reported to mediate autocrine signaling which drives tumor resistance to specific kinase targeted therapy [142].

Oncogenic transformation that is facilitated by paracrine signaling is reflected in the formation of multifocal prostate adenocarcinomas whereby FGF10, which is highly expressed by mesenchymal cells, histologically transforms and stimulates the expression of the epithelial androgen receptor on the adjacent wild type epithelium [143]. In addition, paracrine signaling of FGF10 promoted androgen independent survival of a subset of prostate adenocarcinoma, and also synergizes with epithelial autonomous AKT signaling, leading to high-grade carcinoma [143]. FGF–FGFR paracrine signaling is also identified in a subset of breast cancer stem-like cells. The uncontrolled growth of this subpopulation was proposed to be driven by estrogen hormone stimulation, which in turn, regulates the paracrine signaling of FGF9–FGFR3 [144].

### Impaired negative feedback mechanisms in FGFR signaling

The deregulation of negative regulators of FGFR signaling has been associated with the pathogenesis of various malignancies. For instance, lack of SEF expression has been observed in primary tumors of the breast, ovary, and thyroid [145], while high-grade prostate carcinomas have much lower SEF and SPRY expression compared to healthy individuals [146, 147]. Consistent with this, the levels of SEF is downregulated, whereas FGF2, FGF8, and FGFR4 levels are upregulated in aggressive prostate cancer specimens [148]. Taken together, these data imply that the loss of SEF might contribute to the hyperactivation of the FGF/FGFR signaling axis, thus leading to oncogenesis.

Localization of FGFRs to the surface of the plasma membrane also facilitates FGF ligand binding and intracellular tyrosine kinase transphosphorylation, an event which is required to trigger signal transduction [149]. Under normal circumstances, ligand-receptor complexes are usually endocytosed and transported to lysosomes for degradation. Thus, disruption in the endocytic pathway is expected to alter FGFR signaling [149, 150]. Indeed, in patients suffering from achondroplasia (ACH) and related chondrodysplasia, the substitution of K650E and G380R in FGFR3 protects the surface growth-receptor from being sorted to the lysosomes [151]. The accumulation of these FGFR3 variants, in turn, boosts the signaling capacity of the receptor as their retained half-life is twice that of the wild-type receptor [151].

Similarly, the FGFR4 Arg388 SNP variant which is found in most prostate cancer, has been reported not only to protect FGFR from lysosomal degradation, but also to sustain receptor phosphorylation [125]. Likewise, the FGFR2 IIIb C3 variant which harbors nucleotide deletion at the cytoplasmic C-terminal sequences known to code for the endocytic motif, exhibited aberrant receptor trafficking and stability, and thus, enhanced receptor signaling capacity [152].

### Alternative splicing of FGFRs

Alternative splicing of the IgIII (D3) domain generates the IIIb and IIIc isoforms in FGFRs1–3 [142], and each of these isoforms display different affinity to bind to their FGF family members. For instance, FGFR1 IIIb, FGFR1 IIIc, and FGFR2 IIIc bind FGF2 and FGF9 with high affinity, whereas FGFR2 IIIb preferably binds FGF7 and FGF10 [142].

These IIIb and IIIc isoforms of FGFRs have different roles in cancer. This is reflected in various cancers, including breast, endometrial, cervical, lung, esophageal, gastric, pancreatic, and colorectal cancer [153], which displayed overexpression of FGFR2 IIIb. The role of FGFR2 IIIb, together with its major ligands, such as FGF7 and FGF10, in this context, promotes tumor angiogenesis and migration in pancreatic cancers [154, 155]. However, the tumor promoting roles of FGFR2 IIIb is thought to be exclusive to different cancer types. In line with this, diminished expression of FGFR2 IIIb in gastric cancer cells results in hyperproliferation and invasion [153, 156]. Furthermore, cell lines derived from bladder cancers of lower stage and grade expressed FGFR3 IIIb as their major transcript, while cells derived from high grade tumors exhibit a switch to favour FGFR3 IIIc expression, suggesting that FGFR3 IIIb may have tumor-restrictive properties in bladder cancer [157]. These lines of evidence imply that FGFR2 IIIb and FGFR IIIb play dual roles in tumorigenesis.

Likewise, FGFR1 mRNA is exclusively spliced to generate FGFR1 IIIc in small cell lung carcinoma (NSCLC) cells lines [142]. Additionally, FGFR1 IIIc and FGFR2 IIIc are frequently co-expressed with their complementary ligands—FGF2 and FGF9 in primary NSCLC tumors [142]. The role of IIIc and its respective ligands is thought to compromise a growth factor autocrine loop in a subset of NSCLC cells [142]. FGFR2 IIIc is also associated with the progression of prostate cancers [158], and confers growth advantage to cervical cancer cell lines [159].

Apart from the D3 domain variants, deregulated splicing mechanism affecting other domains are also associated with tumorigenesis. The increased expression of FGFR1β isoform, which lacks the IgI domain, confers increased sensitivity to FGF1, leading to poor prognosis in breast tumors and malignant of astrocytomas [160]. Similarly, a pituitary tumor-derived, N-terminally truncated isoform of FGF receptor-4 (ptd-FGFR4) which lacks the signal peptide and the first two extracellular Ig-like domains, possesses high transforming properties in vitro and in vivo [161]. The targeted expression of ptd-FGFR4 results in pituitary tumors in transgenic mice [161].

In general, the alternative splicing of FGFRs generates multiple isoforms, which are selectively expressed in different types of cancer. These isoforms have tumor promoting and suppressive roles in different cancer types. Further efforts to characterize the novel FGFRs isoforms, and to determine if these isoforms antagonize, or work in synergy to promote or repress cancer cell growth is necessary to improve the design of cancer therapeutics.

Overall, the deregulation of FGFR can occur at several tiers ranging from gene to protein translation and trafficking, and that aberrant FGFR signaling is causally linked to tumorigenesis.

## Current targeted therapies for FGFRs/FGFs signaling pathway

Given the important role of FGFR signaling in tumorigenesis, various approaches have been developed to target the upstream and downstream axis of this signaling pathway. A number of novel therapeutic molecules have been introduced and are currently undergoing preclinical and clinical trials in various FGFRs-related tumors (Table 5). These small molecules can be generally classified as receptor tyrosine kinase inhibitors (RTKIs) which are mainly ATP-competitive molecules; or antagonistic antibodies which target FGF ligands or receptors [162].

**Table 5:** Current status of FGF- or FGFR-targeted inhibitors [19, 165]

Drugs	Target (s) IC_50_ [nM]^a^										Clinical trials	Manufacturer
	FGFR1	FGFR2	FGFR3	FGFR4	VEGFR1	VEGFR2	VEGFR3	PDGFRα	PDGFRβ	Others		
BIBF1120 (Vargatef)	69	37	108	–	34	13	13	59	60	Src: 156 Lck: 16 Lyn: 195	I–III	Boehringer-Ingelheim
TKI258 (Dovotinib)	8	–	9	–	10	13	8	21	27	CSF1: 36 c-KIT: 2	I–III	Novartis
BMS 582664 (Brivanib)	148	125	68	–	380	25	10	–	>6000		I–II	Bristol-Myers Squibb
E7080 (Lenvantinib)	46	–	–	–	22	4	5	51	39	c-KIT: 100	I–II	Eisai
TSU-68 (Orantinib)	1,200	–	–	–	–	2,100	–	–	8	–	I–II	Taiho Pharm.
AB1010 (Masantanib)	–	–	5,500	–	–	–	–	300	50	c-KIT: 150 Lyn: 510	Preclinical	AB Science
AZD4547	0.2	2.5	1.8	164.8	–	–	–	–	–	–	I–II	AstraZeneca
BGJ398	0.9	1.4	1.0	60	–	–	–	–	–	–	I	Novartis

^a^IC_50_ as determined by in vitro kinase assay

*Ab* antibody, *CSF1* colony-stimulating factor 1, *FGF* fibroblast growth factor, *FGFR* fibroblast growth factor receptor, *PDGFR* platelet-derived growth factor receptor, *VEGFR* vascular endothelial growth factor receptor

Most of the RTKIs assessed to date are non-selective FGFRs inhibitors. Despite targeting the FGFR family, they also inhibit other RTKs such as vascular endothelial growth factor receptors (VEGFRs) and platelet-derived growth factor receptors (PDGFRs) [163, 164]. These include compounds like the specific FGFR inhibitor, PD173074; and second-generation FGFR inhibitors that target FGFRs and other RTKs such as dovitinib (TKI258), AZD4547, Ki23057, E7080, brivanib alaninate, intedanib, ponatinib, MK-2461, and E-3810 [162]. Some of these compounds, such as TKI258 and AZD4547 have demonstrated promising potentials as inhibitors of FGFRs, are being tested in advanced clinical trials.

Dovitinib shows high potency against most FGFRs besides targeting c-KIT, CSF-1, VEGFRs and PDGFRs (Table 5) [19, 165]. This molecule is currently being tested in phase III clinical trial for renal cell carcinoma and phase II clinical trials for advanced breast and endometrial cancers, relapsed MM, and urothelial cancer [162]. Given that dovitinib may exert its anticancer effects either by directly targeting FGFRs or regulators of angiogenesis, endometrial cancer patients with or without *FGFR2* mutations are separately enrolled in a clinical to prove its mechanisms of functions [166].

Unlike TKI258 which target multiple kinases, AZD4547 specifically targets FGFR family proteins. Pre-clinical studies have recently demonstrated the selectivity and potency of AZD4547 in FGFR-driven breast tumors cancer models with minimal to none adverse drug reactions at effective doses [167]. In addition, pre-clinical studies on xenograft models transplanted with transformed cells derived from FGFR1-amplified non-small cell lung cancer (NSCLC) patients demonstrated that AZD4547 stops tumor growth and promotes regression [168]. AZD4547 is currently under Phase II clinical trial for breast cancer and phase I for solid tumors [162].

In addition to RTKIs, the development of therapeutic monoclonal antibodies targeting the FGFRs signaling have been demonstrated to exhibit specific antitumor activity in cancer cell lines and animal models. For instance, KM1334 neutralizing antibodies targeting the FGF8 isoform b significantly hinder FGF ligand-mediated signaling in mammary tumorigenesis [169] and FGF8b-expressing clinical prostate cancers [170] in addition to, inflammatory responses and bone damages in rat model of rheumatoid arthritis [169–171]. GP369, an isoform-specific blocking antibody that binds to FGFR2 IIIb have been shown to inhibit the proliferation of several human cancer cell lines and tumor xenografts harboring FGFR2 gene amplification and FGFR2 activating point mutation (S252W and N550K) [99]. R3Mab, an antibody specifically acts on FGFR3 (IIIb and IIIc isoforms) but has no effect on FGFR1, FGFR2, or FGFR4 has been shown to exhibits significant inhibitory effects on FGF1-induced cancer cell proliferation and inhibitory effects on both bladder cancer and MM in mice [172]. Several other antibodies such as IMC-A1 [173], PRO-001 [174], R3Mab [172, 175] and 1A6 [176] also show potentials in treating cancers driven by aberrant FGFR signaling.

Another emerging treatment option is the FGF ligand traps. FGF ligand trap is a fusion of Ig Fc with a soluble FGFR construct that captures FGF1, 2, 3, 7, 10 and inhibits ligand-dependent FGFR signaling [177]. One example of this class of molecule which has been developed as cancer therapeutics is FP-1039, which comprises of the extracellular ligand-binding domain of FGFR1c fused to the crystallizable fragment regions of human immunoglobulin G [178, 179]. The specificity of this ligand is reflected by the observation that the growth rate of head and neck squamous cell carcinoma cells expressing abundant FGF2 is significantly inhibited upon treatment with FP-1039, whereas HNSCC cells that express little FGF2 is not affected [179]. FP-1039 is currently in a Phase II trial for endometrial cancers with the S252W *FGFR2* mutation which confers increased affinity and altered specificity of FGF binding [178].

Other strategies targeting the FGFR signaling such as peptide mimetics, RNA aptamers, siRNAs and miRNAs have also been investigated intensively merits further improvements [180].

## Challenges in the targeting FGFRs in human cancers

Given the broad expression of FGFRs and their key role in development and physiology, toxicity issues are to be expected from FGFR inhibition. The FGFR pathway is involved in normal phosphate and vitamin D homeostasis. The preclinical development of FGFR inhibitors has been complicated by hyperphosphatemia-mediated tissue calcification, due to the blockade of FGF23 release from bone and of FGF23 signal in kidney [181]. In preclinical models, dynamic modulation of circulating FGF23 levels was reported following FGFR inhibition. FGF23 levels was suppressed during the periods of drug exposure attributed to direct inhibition of FGF23 release from the bone and elevated upon drug withdrawal driven by increased plasma phosphate and vitamin D levels acting on bone to stimulate FGF23 production [182]. FGF23 may also bind FGFR4, FGFR1 IIIc and FGFR3 IIIc [183, 184], but the relative contribution of individual FGFR subtypes to hyperphosphatemia remains unclear [185–187].

Similarly, administration of an anti-FGFR1 IIIc antibody has been shown to result in profound weight loss in preclinical in vivo models, potentially due to FGFR1 targeting in the hypothalamus [173], and this has prevented further clinical development. It remains to be ascertained whether this would be a class effect for all FGFR1 IIIc antibodies.

Finally, the great challenge for future development of specific FGFR inhibitors in the clinic is to carefully determine a therapeutic dose which will balance efficacy against gene-addicted tumors with a manageable tolerability profile. Continued clinical research may identify which FGFR isoforms have the greatest efficacy potential, and whether inhibition of particular isoforms can avoid side effects associated with broad specificity small-molecule FGFR inhibitors.

## Conclusions

In conclusion, the role of FGFRs signaling in promoting tumorigenesis is well established. As such, novel molecules which inhibit FGF or FGFR interactions have been introduced and many of them are currently undergoing clinical trials for treating various types of human malignancies associated with hyperactivation of FGFR signaling. Given that the standard chemotherapies in cancer patients are often associated with adverse toxicity, such targeted-therapies could present a more viable option in addressing this issue. In addition, the inhibition of molecules downstream of FGFRs network could also serve as a secondary approach as many of these molecules regulate various cellular processes and functions as well. With the emergence of drug resistance and disease recurrence issues, the current trend of clinical approach is moving towards multi-targeted drugs and combination therapies. This requires in-depth understanding of the signaling pathways in order to formulate an appropriate strategy which focuses on cancer cells. The clinical application of these therapeutic strategies warrants further studies to ensure maximum clinical benefit in cancer patients.
